# Clinicians’ Decisions to Screen for Intimate Partner Violence Use and Experience and Observed Impacts: Qualitative Study

**DOI:** 10.2196/81651

**Published:** 2026-05-29

**Authors:** Sarah A Walls, Julie D Yeterian, Skye Orazietti, Candice Presseau, Galina A Portnoy

**Affiliations:** 1 Research Department, VA (Veterans Affairs) Connecticut Healthcare System, 950 Campbell Avenue, West Haven, CT, 06516, United States, 1 619-302-9234; 2 Department of Psychiatry, Yale School of Medicine, New Haven, CT, United States

**Keywords:** screening, intimate partner violence, health care, quality improvement, veterans

## Abstract

**Background:**

Recent research has found that concurrent intimate partner violence (IPV) experience (ie, victimization) and use (ie, perpetration) may be more common than experiencing or using IPV in isolation. Therefore, screening for IPV experience and use concurrently is needed to provide resources and connect patients to care.

**Objective:**

In this work, we explore how clinicians made decisions to use a screening protocol for IPV use and experience and their perceptions of how concurrent screening impacted patients, clinicians, and the health care system.

**Methods:**

We conducted qualitative interviews with 19 clinicians (18 women and 1 man) who participated in a 90-day pilot screening implementation initiative, in which they were asked to integrate screening for IPV use and experience concurrently into their daily practice. Most clinicians (17/19, 89.5%) had prior IPV-related training. Interviews focused on clinicians’ experiences implementing the screening tool and were analyzed using thematic analysis.

**Results:**

We identified four themes: (1) new screening implementation is challenging, (2) screening for IPV use and experience concurrently can be uncomfortable, (3) pivoting strategies can make screening easier, and (4) screening for IPV use and experience concurrently is impactful. Findings highlighted complexities of implementing new screening protocols, as clinicians spoke about the importance of screening for IPV use and experience concurrently, while pointing out barriers to integrating the screening protocol into their daily clinical routines. Clinicians made adaptations to the screening protocol and the screener itself to assist with adherence to screening efforts.

**Conclusions:**

Findings demonstrate the need for and importance of screening concurrently for IPV use and experience, while bringing awareness to difficulties with implementing any new screener. Findings underscore the importance of addressing barriers to increasing screening efforts for IPV use and experience concurrently through increased allotment for screening efforts. The results also highlight opportunities for pivoting strategies and ongoing training and education around managing concurrent IPV use and experience. Future research should explore how decreasing barriers to screening efforts and adaptations to screening practices impact decisions to screen, while also exploring clients’ perspectives on being screened for IPV use and experiences concurrently.

## Introduction

Reported rates of past-year intimate partner violence (IPV) experience (ie, victimization) and use (ie, perpetration) are comparable, impacting around 6%‐7% of the US population [1,2]. Health care systems have historically focused solely on screening for IPV experiences, specifically for women under 48 years old [3-5]. However, concurrent IPV use and experience (sometimes called bidirectional or mutual IPV), in which both members of a couple use aggression or violence (ie, emotional, verbal, physical, or sexual) during the same or separate episodes, is more common than IPV used by 1 member of a couple [6-10]. Limiting IPV screening to experiences of violence only may result in missed opportunities for detecting other forms of IPV, thereby potentially limiting awareness of and access to violence prevention and intervention services and resources [11]. By implementing screening protocols to assess IPV use and experience in tandem for all patients, health care systems can optimally support early detection and connection to care. This study, conducted with clinicians who were trained to administer IPV use and experience screening concurrently in a large health care system, examined factors that influenced their decisions of whether or not to screen and their perceptions of the impact of conducting concurrent screening.

Conducting routine, ongoing screening for IPV increases the likelihood that a patient will receive prevention or intervention services [5,12]. However, screening rates for IPV experience vary widely across studies and contexts. The median screening rate in research studies examining IPV experience screening programs appears quite high at 80% of eligible women [13], whereas rates in routine clinical practice are often lower (9.5% of women screened) [3,14]. Further, a minority of clinicians conduct universal or near-universal screening within their clinical practice (2%‐50%) [15]. A systematic review found that veterans have higher rates of IPV experience and use than the general population, with 24.3% of veterans experiencing IPV and 31.8% of veterans using IPV [14], highlighting the need to focus on screening practices within the Veterans Affairs (VA) health care system. IPV screening within the VA health care system’s primary care has increased considerably, from fewer than 500 patients in 2014 to nearly 35,000 in 2020 [3]. Despite the notable increase, those being screened represent only 17% of women primary care patients at sites that have implemented screening [3].

Although an understudied area, available data suggest that screening for IPV use is far less common than screening for IPV experience [16,17]. A recent scoping review on IPV use screening and identification within hospital systems found an absence of formal mandates or protocols for identifying people who use IPV, which may lead to inconsistent, nonsystematic screening and referral practices within and across settings [18]. Additionally, 1 study of IPV use screening practices within the VA health care system found that a minority of male patients with posttraumatic stress disorder (24%) had a documented screening for IPV use [5]. Yet, additional research has shown that when screening for IPV use does occur among veterans participating in mental health treatment, nearly half of men and women report using some form of IPV in the past year [8].

There are numerous reasons why IPV screening may not be completed even when recommended or mandated within a health care system. For instance, medical clinicians may believe they lack the training or competency for screening for IPV [4,19] or that screening is outside the scope of their discipline [20,21]. Clinicians may also feel uncomfortable or unsafe asking patients about their IPV experience or use [22-24]. Systems-level issues such as limited staffing, concerns around safety, and insufficient time for screening can also influence clinicians’ decisions to screen for IPV experience [4,13,20,25,26].

Conversely, clinicians are more likely to screen when there are clinic policies for screening, when patients self-disclose, or when clinicians believe a screen is warranted, and/or during intake sessions [21,27]. Having access to IPV resources can increase clinicians’ willingness to screen [21]. Prior work has shown that patients are willing to be asked about and disclose their IPV to health care clinicians, particularly clinicians with whom they feel comfortable [16,28,29].

In response to the emergent need for improved screening and response for IPV use, clinical practice guidelines were developed to address IPV use disclosures in health care settings [30]. A natural extension of this work is to explore how clinicians decide who and when to screen for IPV use and experiences concurrently and their perceptions of the impact of concurrent screening on patients, clinicians, and health care clinical settings more broadly.

Concurrent IPV use and experience is quite common across veteran and civilian relationships [7,31-34], with a recent scoping review finding the median rate across 34 studies to be 35% vs 17.5% for unidirectional IPV [10]. It is crucial to understand clinicians’ experiences screening for IPV use and experience across patient populations. This study analyzed qualitative interviews of 19 mental health clinicians who participated in a pilot focused on implementing screening for IPV use and experience concurrently in routine mental health care. This study aimed to examine how clinicians made choices about screening for IPV use and IPV experience concurrently, as well as their perceptions of the impact of screening on patients, clinicians, and the health care system.

## Methods

### Study Design

Data for this analysis were collected as part of a quality improvement (QI) project evaluating the implementation of an IPV use and experience screening protocol for veterans accessing mental health services at 6 VA medical centers [8]. The screening protocol included (1) a 10-item screening tool for past-year IPV use and experience, the Relationship Violence Use and Experiences Scale; (2) procedures for responding to positive screens; (3) safety planning procedures; (4) guidelines for providing universal education, resources, and referrals; and (5) medical record documentation guidelines [8]. Consistent with VA Directive 1198 [35], the screener was only administered when the veteran was in a private space (ie, was alone) and consented to complete screening.

Clinicians from 6 medical centers self-selected to participate in the QI project. Participating clinicians received training to administer the screening protocol and were asked to implement screening in their routine clinical practice during a 90-day pilot window, while receiving ongoing support and consultation from site leads and project leadership. While fidelity to the screening protocol was not formally assessed, challenges that clinicians experienced in administering the screener were discussed and problem-solved during consultation calls. Following the 90-day screening period, clinicians had the option to participate in a semistructured qualitative interview about their experiences implementing the screening and response protocol. This report follows the COREQ (Consolidated Criteria for Reporting Qualitative Research; Checklist 1) [36].

### Ethical Considerations

This evaluation is a QI initiative jointly supported by the VA Care Management and Social Work Service’s IPV Assistance Program and VA Connecticut Health Care system [37]. This initiative was designed for internal purposes in support of VA QI as an internal operations evaluation and was designated as nonresearch by VA, thus not requiring institutional review board approval. Clinicians voluntarily agreed to be interviewed and were not compensated. Verbal consent was obtained before commencing the interview recording process. All identifiable information was removed from transcripts before analysis.

### Sampling and Participants

A total of 32 clinicians (including 1 site lead at each of the 6 sites) in various clinic settings (ie, mental health, homelessness services, and primary care) participated in the screening implementation QI pilot and were eligible to participate in qualitative interviews following the pilot period. Further, 5 clinicians were not eligible to participate in the interviews after withdrawing from the project or leaving their positions. This study’s team invited the remaining clinicians to participate in qualitative interviews via email and conducted individual interviews with every clinician who responded to email outreach (n=19, 70% of those eligible). Reasons for nonparticipation from those who did not respond (n=8) were not collected. We used an information power approach to sampling [38]. Following this approach, there were several aspects of our study that allowed us to address our research questions using a relatively small sample. These included our study’s relatively narrow aims, conducting interviews with individuals with a highly specific set of experiences related to those aims (ie, experiences with screening practices as guided by VA Directive 1198 [35]), implementing a new screening tool and procedure for IPV use and experience, use of foundational theories to create the interview guide, interviewers with extensive knowledge of IPV and the QI project, and a rigorous, systematic approach to data analysis.

Table 1 presents the demographic characteristics of participants; participants who opted out (n=8) were similar to those whom we interviewed, in demographics and clinical training. Participants categorically reported seeing a high volume of clients per week, with the majority (9/19, 47.4%) seeing an average of 20 to 39 clients in 1 week, while others reported seeing 10 to 19 (6/19, 31.6%) and less than 10 (4/19, 21.1%). The majority of participants had received IPV-related training prior to starting the pilot (17/19, 89.5%). Virtually all (18/19) participants reported previously screening their patients for IPV experiences, and 14 reported previously screening their patients for IPV use.

**Table 1.** Individual characteristics of Veteran Health Administration clinicians interviewed after implementing a concurrent IPV^a^ use and experience screener (N=19).

Characteristic	Participants, n
Gender	
Men	1
Women	18
Discipline	
Social work	14
Psychology	2
Psychiatry	1
Pharmacy	1
Advanced practice nursing	1
Age group (years)	
18‐29	3
30‐39	6
40‐49	5
50‐59	3
60 or older	2

a IPV: intimate partner violence.

### Data Collection

Semistructured interviews were developed using the Theoretical Domains Framework [39] and the COM-B (Capability, Opportunity, and Motivation framework of Behavior change) [40], implementation science theories and frameworks that propose specific determinants of or essential conditions for behavior change. Interview questions covered overall impressions; perceived barriers to and facilitators of screening; knowledge, skills, and beliefs about capabilities; screening in relation to social or professional role and identity; consequences of screening; and the relation of emotions to screening. Examples of questions included “How did you decide when and who to screen?” “Do you plan to continue using the bidirectional IPV screener or any other parts of the package?” “Why or why not?” “What factors made you most likely to conduct screening during a particular clinical encounter or session?” and “How did incorporating the screener impact your workflow?” Interviewers used follow-up prompts (eg, “How so?” “Tell me more about that”) as needed to encourage respondents to elaborate on brief responses.

Semistructured interviews were conducted and recorded using Microsoft TEAMS. On average, interviews were conducted 28.5 days (mean 28.55; SD 12.42; range: 9‐51) after the end of the 90-day screening window. The interview team included 5 women with social work and/or psychology backgrounds, including 3 of this paper’s authors (SAW, SO, and GAP). Interviewers were trained on the use of the semistructured interview over the course of 2 meetings in which they piloted the interview guide. Interviewers also engaged in a reflexive practice before each interview, informed by Hsuing [41]. This self-reflective exercise asked interviewers to examine potential biases, assumptions, and emotions related to IPV experience and use within relationships. Further, 2 interviewers were present at each interview, with 1 conducting the interview and the second taking notes to be used during debriefing. Furthermore, 2 of the interviewers were known to the participants as they provided training and background information on the development and administration of the screener.

At the start of each interview, interviewers provided a verbal overview of the process, including the estimated time commitment (≈45 min), the voluntary nature of the interview, and the purpose of the interview (ie, to inform modifications to the screening materials and implementation strategy). Interviewers shared a brief overview of the screening project at the start of the interview. Interviews lasted 39 minutes on average (mean 39.23; SD 6 min; range=28‐51 min). Immediately after each interview, the interview team met to debrief by discussing the interview, noting initial impressions, and exploring possible modifications to the interview (eg, shifting the order of questions). Interviews were recorded and transcribed verbatim. Participants were not compensated for their participation.

### Data Analysis

Using a team-based approach, 3 coders (SAW, JDY, and SO) analyzed the transcripts following the 6-phase approach to thematic analysis (TA) by Braun and Clarke [42,43]. We used a mixed inductive or deductive approach to coding due to the semistructured nature of the interview guide and to allow themes to emerge organically through the systematic approach provided by TA [44]. Our coding was guided by two research questions (deductive): (1) How did clinicians make choices about screening for IPV experience and use concurrently? and (2) What was the impact of screening for IPV experience and use concurrently on veterans, clinicians, and systems? Within this general framework, however, we had no preconceived codes or themes and coded all content that could relate to our research questions (inductive). We selected TA as our data analytic approach because it allowed us to systematically identify patterns in participants’ responses that were relevant to our research questions while also providing a framework for moving beyond these rich descriptions into an interpretation of how themes were interrelated [42,43]. While interview guides were developed using the Theoretical Domains Framework [39] and COM-B [40], we opted to use TA for data analysis to take a more inductive approach and because our second research question was not directly related to behavior change.

Before coding each transcript, coders read the transcript in its entirety and noted initial ideas about possible themes and relevant responses (phase 1). The coding team independently coded transcripts manually, line by line, and met to review codes and coding styles in an iterative process. The team created the codebook through this process, identifying codes that appeared across multiple interviews, organizing codes by research question, and refining the codebook after applying it to subsequent transcripts. The final codebook included 21 codes across the 2 research questions (phase 2; see Table 2).

**Figure 1.**
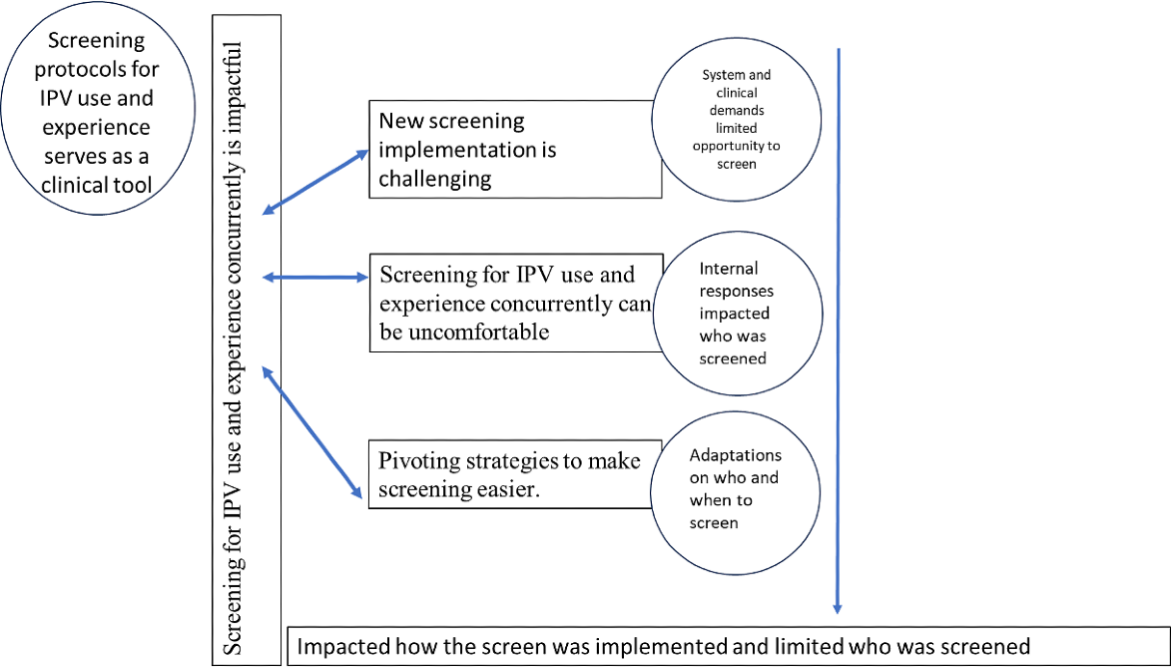
Thematic map of the interrelationship between themes from interviews with Veterans Health Administration clinicians (N=19) about implementing a concurrent IPV use and experience. IPV: intimate partner violence.

Each member of the team independently identified possible themes represented by the 21 codes, followed by a meeting of the full team in which we collaboratively discussed these possibilities and worked to integrate our perspectives. This led to the development of 4 initial themes, collapsed across the 2 research questions (phase 3). We then reread the coded transcripts with these themes in mind, working to evaluate whether the themes were aligned with the raw data and to refine the themes accordingly, both individually and as a team (phase 4). Finally, we defined and named our themes (phase 5), including creating a thematic map of the relation of codes to themes (Figure 1).

**Table 2.** Final thematic codebook derived from interviews with Veterans Health Administration clinicians (N=19) about implementing a concurrent IPV^a^ use and experience screener.

Research question	Code
Q1: How did clinicians make decisions about screening for IPV experience and use concurrently?	Screening based on perceived relevance Need to balance competing priorities Desire to have more time to screen Concerns around screening via telehealth Need to adapt to circumstances or population Deciding not to screen without veteran consent Clinician being uncomfortable with screener and/or screening process Not wanting to ask people who are referred for IPV experiences about IPV use Everyone was supposed to be screened Deciding to screen during intake Deciding to screen an easy-going client
Q2: What was the impact of screening for IPV experience and use concurrently on veterans, clinicians, and systems?	Difficulty containing responses Screening affects quality of life at work Screening picks up IPV cases that might be missed Screening educated veterans and clinicians about IPV Veterans accepted screening Screening brings up shame for the veteran Veterans had negative response to screening Screening facilitates additional treatment connections Impact on system What clinicians need as individuals

a IPV: intimate partnet violence.

## Results

### Overview

We identified several themes relating to clinicians’ decisions to screen for IPV use and experience concurrently, and observations of the impact of screening on patients, clinicians, and the health care system. Themes highlighted the tension between clinicians’ desire to screen for concurrent IPV use and experience and the complexity of implementing screening within large health care systems. These themes were as follows: (1) new screening implementation is challenging; (2) screening for IPV use and experience concurrently can be uncomfortable; (3) pivoting strategies can make screening easier; and (4) screening for IPV use and experience concurrently is impactful. Figure 1 shows the interrelationships between these 4 themes.

### New Screening Implementation Is Challenging

Clinicians expressed numerous challenges related to implementing a new screening tool and highlighted the overwhelming nature of screeners in large hospital systems in general. While acknowledging the importance of screening, they described having to implement it as yet another task to be completed with limited time in the appointment, and described the time required as a significant consideration in their decision of whether or not to screen.

*This never ending like, okay, here’s 10 new … screenings that you have to do. It’s just hard to balance all of them.*

[Interview #3, p.6]

*I felt that I often was hesitating to do the screen—well, first of all, I was hesitant to do the screener with people if I thought that I wasn’t gonna be able to… have the time—enough time to devote to it to really give it what it deserves.*

[Interview #17, p.3]

Respondents spoke about the difficulty of balancing screening with competing clinical demands.

*My best efforts, it just became this 15-minute thing and then I had almost no time left to do anything else. Which when that’s the presenting concern, works out really well because I’m getting rich information that’s really valuable. But when it’s not the main presenting concern, then it’s sort of taking away what could be—what really important time for the Veteran to be—for me to be evaluating them and coming up with a plan.*

[Interview #1, p.7]

Clinicians also reported that, given the time demand for screening, they encountered difficulties completing all of their assigned tasks. For example, clinicians, aiming to screen everyone, mentioned having to work during their lunch break, not having a chance to use the bathroom, and working past their expected hours. Such experiences, in turn, impacted how the screener was implemented (eg, clinicians diverging from the protocol, such as by shortening the screener) and influenced clinicians’ decisions about who to screen (eg, when it was perceived as “easy,” such as an anticipated negative screen outcome).

Participants described needing additional support and resources (both at the individual and systems levels) to screen effectively and sustainably. They noted wanting to continue to provide screening after the end of the pilot period and stressed the importance of leadership support for this:

*So I do think going forward beyond a pilot and rolling this out, I do feel like leadership support and investment or backing of the screen and the importance is gonna be needed.*

[Interview #12, p.5]

Along with leadership support, clinicians identified strategies to continue screening or to ensure screening was feasible, such as shortening the screener length, having a paper version, and ongoing training for clinicians administering the screener and for leadership (to increase awareness).

### Screening for IPV Use and Experience Concurrently Can Be Uncomfortable

In addition to the challenges of implementing a new screening tool, respondents identified concerns that were specific to the IPV use and experience screener. Clinicians shared concerns about what the screening was like (or would be like) for them and their patients. For example, clinicians expressed feeling anxious or awkward about the screening process, such as asking questions about IPV use when they knew a veteran had experienced IPV.

*Well, this is a minor thing, but I felt weird when I had Veterans refer to me—when I knew that they were experiencing violence, like that’s why they were referred to me. And this just might be ‘cuz I’m in IPVAP [VA IPV program that provides support and resources for IPV], but it felt weird to me then to start the screen with asking about use of violence (Interview #2, p.2)…I didn’t do it on ones that were coming in with very clear experience of violence. Like they came in with like significant things that they’re going through. And so, I didn’t wanna then say, and are you doing that to your partner as well? It didn’t feel very validating.*

[Interview #2, p.6]

Some clinicians raised concerns about screening via telehealth, such as other family members being present or the lack of a private place to talk, and noted that screening would be easier during an in-person visit, where the environment was easier to control. Other clinicians used clinical judgment when deciding who to screen and shared a desire to avoid “shame” responses from their patients. These concerns were underscored by the negative reactions that they reported from their patients, thus impacting their decision-making in the screening process:

*There was one who said that the questions made him uncomfortable, particularly the question about sexual abuse to others and forcing and that sort of thing. He said it made him feel icky to hear that question because he had never considered anything like that.*

[Interview #4, p.5]

### Pivoting Strategies Can Make Screening Easier

As illustrated in the previous 2 themes, respondents noted that screening all patients in their routine mental health practice was difficult to implement. The third theme describes how clinicians used a variety of adaptations and strategies to fit screening into their current practice, making pivots or changes that allowed them to screen. These methods included selective screening based on perceived relevance and/or the cooperativeness of the patient (instead of screening all patients), adaptations to the procedure, and integrating screening into the intake process. For example, clinicians used presenting clinical concerns as an indicator to administer the screener:

*If I knew there was some hint of IPV, then I would preempt the regular therapy to do the screening.*

[Interview #5, p.6]

Others used established rapport and/or knowledge of the patient (eg, their relationship status) to make decisions regarding screening:

*I would typically decide who based off of their temperament in the session. If they were a little bit more easy-going or open to like different parts of the …conversation, I would definitely do it.*

[Interview #14, p.7]

*I don’t know if this is maybe because if I didn’t have time—these are patients I know really well and I know that this is something that’s not applicable then I’m not—then I was not doing it…I just wasn’t doing it if I thought that it wasn’t applicable or if like someone that is like not at all in a relationship or it just didn’t seem like—I have a lot of 75-year-old Vietnam Veterans who live by themselves…But if I did suspect that—or if I had an idea, or they were in a relationship, but I wasn’t sure, I didn’t know, I did try to make my best effort to do that.*

[Interview #17, p.5]

Clinicians adapted the screener in different ways to facilitate implementation. Many clinicians pointed out that they used portions of the screener or made adaptations to the screening protocol. For example, 1 clinician stated:

*I would actually do it paper/pencil because it went quicker for me.*

[Interview #12, p.3]

Another specified a preference for patients to complete a self-administered screener before the appointment, with the intent to facilitate conversation based on screening results. Others noted that they tended to integrate the screener into their intake process to make it feel more natural or because they had more time with the patient.

### Screening for IPV Use and Experience Concurrently Is Impactful

Despite challenges, clinicians found value in screening and highlighted the need for, and importance of, an IPV screener for experiences and use concurrently. Clinicians described the importance of creating an approach to screening for IPV where patients felt safe to explore, learn about, and disclose IPV. They emphasized that screening for IPV use and experience concurrently provided the opportunity for conversation, communicating to patients that clinicians are interested in all aspects of health and wellness, including the often-hidden aspects of home life. As a result, screening could facilitate a nonjudgmental approach to asking about IPV use, creating a safe space for disclosure and opportunities to respond and connect those who use IPV with resources.

*I do think that was one of the strengths that allowed the conversation for me and the Veteran to have. And I think it also gave, it gave—oh gosh, it gave me in this role another kind of lens to look out of in primary care that might not often be looked at.*

[Interview #14, p. 2]

Clinicians acknowledged that even when screening concurrently resulted in a negative screen, the process opened doors to conversation, resulting in patients feeling safe to bring up the topic when ready. Further, 1 clinician shared wanting to “go back” and screen again to continue an important conversation:

*There are a couple of other people that I want to now go back to the screener and go through it again with them because there’s one person in particular who had always indicated that his ex-wife had been a chaotic and difficult person, but after we did the screener, and he didn’t identify really in either direction that there was physical or any kind of extreme abuse, but in conversations afterwards, he has brought up how abusive she was to him and that he attempted suicide when she asked for a divorce. Just really interesting stuff. So maybe it takes time for it to sink in.*

[Interview #8 p.7‐8]

Clinicians emphasized the novelty of asking patients about IPV use (vs IPV experience alone), furthering insight into patients’ experiences and possible impacts on their health and wellbeing.

*So, one, it worked primarily with our women Veterans and recognizing that there’s certainly a high proportion of my Veterans who experience violence but noticing that I never really thought about asking about the use of violence aside from our kind of general SI, HI questions that we ask but nothing beyond that. So, just curious to kind of see from the bidirectional standpoint what might be reported and what I might have been missing by not intentionally asking these questions.*

[Interview #19, p.1]

Respondents also mentioned how the screener serves to educate patients and clinicians about what behaviors constitute IPV (eg, yelling), which then picks up cases of IPV that might otherwise have not been detected, and provides context to the broad definition of abuse for both clinicians and their patients.

*I had actually a couple of people that recognize that there were some unhealthy behaviors going on in the context of the relationship. But due to stigma or due to the way that cultural values or norms views violence, that if it’s not physical then it doesn’t have any negative implications. So, I think there were a couple of moments when people had some good awareness of, all right, it’s more than that. Maybe what’s going on isn’t healthy in my relationship and they were able to gain that because of going through the screening.*

[Interview #19, p.9]

Clinicians described the benefits of screening for their patients and patients’ partners, families, and home life. They described how screening helped to facilitate connections to appropriate treatment resources.

*I had a Veteran that I screened and got connected to our IPV program... And his kids—there was a CPS [Child Protective Services] call that I had to make as well, and his kids are back in his custody. I know that his part of the CPS recommendations were to get connected to treatment and to services and he did that and he’s following everything that they have asked. And from what I know, he’s doing really well. So, I think, that alone was a huge win.*

[Interview #16, p. 8]

## Discussion

### Principal Findings

This study explored clinicians’ decisions to screen for IPV use and experience concurrently. Decisions about when to screen and how to administer the screening and response protocol were made based on logistical (eg, time and resource constraints) and clinical (eg, past IPV experiences; patient’s style) considerations. We also found that despite barriers to implementing an IPV use and experience screening tool and response protocol, clinicians recognized positive impacts of screening. Clinicians raised concerns that could apply to any new screening process, as well as some that were specific to screening for IPV use and experience.

Identified barriers (ie, time restrictions, length of screener, or desire for leadership support) are consistent with prior work on implementing IPV screening [4,13,19,20,25]. Clinicians described discomfort with screening for IPV experience and use concurrently, such as perceived awkwardness and concerns around shaming their patients. Notwithstanding these challenges and the unintended consequences (ie, working through lunch or not being able to complete all assigned tasks), clinicians indicated that screening for IPV use and experiences concurrently helped patients open up about their home life and develop new insight into potentially harmful relationship behaviors (eg, yelling). In turn, clinicians were more able to assist them in getting connected to resources and follow-up care. Clinicians also described strategies they used to tailor the screening protocol to manage these challenges, such as by selectively screening particular patients. Prior research has highlighted how practical limitations often lead to selective screening vs universal screening practices [15].

This work has important clinical and policy implications. The tension between the importance of universal screening and the reality of putting that into practice within a large health care system highlights that system-level changes are needed to ensure the uptake and sustainability of screening for IPV use and experience in routine practice. For example, identifying and training clinicians to screen comprehensively for IPV, creating more time for screening during clinical encounters, and prioritizing time for the screening process during appointments were factors identified within the local system that would support screening. In addition, further training, increased awareness of resources and interventions among all staff (not just those who provide the screening), and greater leadership support could strengthen and expand screening efforts. Leaders could ease the burden on clinicians by decreasing clinical demands or increasing the length of appointments to integrate screening efforts, potentially allowing clinicians to increase screening rates for IPV experience and use concurrently.

Screening may not only assist with connecting patients to appropriate follow-up care, but can also provide opportunities for education, both of which serve as primary prevention strategies [45] and can prevent escalation of subsequent, possibly more severe, violence within the relationship. Prior research has shown that clinicians may have a level of discomfort and a perceived lack of experience when screening for IPV experience in women [46]. Clinicians who piloted the current screening and response protocol for IPV use and experience expressed similar sentiments. Specifically, they shared concern around screening for use of violence when the patient also experienced violence, highlighting the need for more awareness of how common bidirectional IPV is, particularly in veteran relationships [34].

Since early research regarding IPV screening practices, clinicians’ awareness, training, and comfort with IPV experience screening have increased [5,24,47]. Based on these efforts, we surmise there would be a similar trajectory for implementing screening for IPV use and experience concurrently in health care settings. Indeed, a cohort study of veteran men with posttraumatic stress disorder found that when screening for IPV use was documented in health records, other clinicians were more likely to screen for IPV use [5]. Further, when the veterans were screened more than once, their level of self-disclosure of IPV use was higher.

A scoping review of IPV use disclosure and outcomes found that nurses and other clinicians, similar to the clinicians interviewed for this study, used clinical indicators such as presenting concerns (eg, substance use or injuries) as indicators to prompt screening for IPV use [18]. Some clinicians in our study identified feeling uncomfortable with screening veterans out of concern that it might not be relevant to them or that they might react negatively. Other research has found that clinicians are more likely to screen when patients self-disclose IPV or during intake [21,24], suggesting that there may be particular conditions under which screening feels more natural, a theme echoed in our qualitative interviews. Research has found that providing IPV training for clinicians about those experiencing IPV can increase clinicians’ rates of screening and confidence with disclosures [45]. As such, and with consideration for the clinicians’ expressed preferences for additional training to support broader implementation, offering brief training opportunities through accessible means (eg, prerecorded training) would likely serve to increase comfort with screening for IPV use and experience concurrently, especially in earlier phases of implementation.

Although there is some empirical support for the potential benefits of universal screening for IPV experience in routine practice (eg, overall improved psychosocial support [26], education about IPV [48], detection [26], and improved trust and rapport with clinicians [49]), our results indicate that universal screening for IPV use and experience concurrently may not be the preferred approach among clinicians, even those who recognize its importance and value. Yet, routine screening for IPV use and experience concurrently may provide the opportunity to develop a foundation of trust for possible future disclosures, as has been found in research for IPV experience [50] and IPV use [51]. Screening for IPV use and experience concurrently and routinely also allows clinicians to capture the full experiences of their patients. Given the systems-level difficulties clinicians encounter (eg, time or competing priorities), universal screening may not be the most feasible approach, nor is it yet indicated without additional research and evaluation. Additional training and leadership buy-in may offer some avenues for improving implementation; however, they may not entirely mitigate the real-time decisional challenges that clinicians face (eg, additional presenting concerns that require attention).

### Implications for Future Research

Additional work is needed to evaluate the relative effectiveness of adapted or innovative screening practices to balance the needs and experiences of clinicians with the needs of the patients they are treating. A consideration for further exploration is to assess collaborative approaches to the screening protocol by prioritizing education about IPV experience and use before engaging in screening practices [52], which may decrease barriers and increase awareness of the need for and importance of screening for IPV use and experience concurrently. Although IPV use and experience are more prevalent at higher rates within the veteran population [14], IPV is a public health issue, and implementing any new clinical practices is challenging within large health care systems [4,13,19,20,25]. As such, we believe the results can be broadened to nonveteran serving health care systems [25] and be used to inform the development of scalable, sustainable IPV screening programming that attends to concurrent IPV use and experience. For example, anticipating clinicians’ challenges and limitations (eg, lack of time or competing priorities) and ensuring they receive sufficient guidance and leadership support for procedures that are appropriately adapted to their patient populations and clinical settings (ie, increased appointment time for new screening requirements or acknowledgment of effort) [53] will be crucial to future initiatives and expansions of this work. Lastly, future exploration around adaptations made by clinicians to the screener and the screening protocol, and how such changes may impact their decision-making in screening for IPV use and experience concurrently, is needed to increase awareness of the feasibility of screening practices.

### Limitations

This study has several limitations. Clinicians self-selected to participate and were likely intrinsically motivated to implement a screening for IPV use and experience in their practice. Social desirability bias may also be a consideration, as 2 of our interviewers (SO and GAP) were involved in developing the screener (GAP), training the clinicians on the screener (GAP), and were engaged in weekly administrative communication (SO). As such, their presence may have influenced clinicians’ stated opinions about the benefits and perceived value of the training. Decisions to screen may look different among clinicians with different backgrounds or who are screening as a result of a health system policy requirement. Future research can examine IPV use and experience screening implementation in different settings and contexts (eg, beyond mental health services; as part of a clinic mandate). Second, the 90-day period for the pilot may also have limited the opportunity for clinicians to implement screening into their routine clinical practice, as they had only a relatively brief period in which to observe factors that influenced how decisions about screening impacted their clinical flow. Thus, our participants could not meaningfully comment on how the screening impacted clinical outcomes over the long term, and whether their decision-making processes were influenced by experiences gained over a longer period of time. It is unclear whether similar results would have been found if screening had been implemented over a longer period.

An additional limitation to our research is that we did not collect data from patients themselves on their experiences of screening. Similarly, none of our clinician participants mentioned formally assessing their patients’ reactions to the screening. While some prior research exists on how patients think they would feel about being screened for IPV use and experience concurrently [16], the perspectives of patients who have directly experienced this type of screening are needed to fully capture the impact. For instance, our clinician participants at times reported on their patients’ reactions (eg, recognizing unhealthy behaviors within their relationships or discomfort with some screening questions) or anticipated reactions (eg, shame or invalidation). Talking to patients who were screened directly about their experiences could confirm and/or refute these clinician perspectives.

### Conclusions

Implementing new screening tools and processes poses challenges within health care systems [54-57]. Thus, it is not surprising that clinicians encountered challenges screening for IPV use and experience concurrently during this implementation pilot. Our findings underscore that screening for IPV use and experiences concurrently has the potential to serve as an important prevention strategy in health care settings and is perceived as beneficial by clinicians. Clinicians identified practical recommendations for promoting screening, including additional training and increased leadership support that warrant further study. Further research is recommended to (1) investigate the capacity of such methods to improve the adoption of screening for IPV use and experience concurrently across diverse health care systems, (2) track adaptations clinicians make and integrate needed changes into the screening protocol, (3) investigate implementation strategies needed to increase clinician screener usage, (4) explore patient perspectives of being screened for IPV use and experiences concurrently to determine necessary improvements and innovations to enhance their health care experiences, and (5) investigate the effectiveness of adapted and/or selective screening approaches using the integration of quantitative metrics such as the numbers and percentages of patients who receive IPV-related services following screening and clinician and patient satisfaction ratings following clinical encounters with these alternative approaches.

## Supplementary material

10.2196/81651 Checklist 1 COREQ checklist.

## Abbreviations

COM-B: Capability, Opportunity, and Motivation framework of Behavior change
COREQ: Consolidated Criteria for Reporting Qualitative Research
IPV: intimate partner violence
QI: quality improvement
TA: thematic analysis
VA: Veterans Affairs
